# The surgical management of locally advanced well-differentiated thyroid carcinoma: changes over the years according to the AJCC 8th edition Cancer Staging Manual

**DOI:** 10.1186/s13044-019-0071-3

**Published:** 2019-10-27

**Authors:** Alessio Metere, Valerio Aceti, Laura Giacomelli

**Affiliations:** Department of Surgical Sciences, Umberto I Hospital, “Sapienza” University of Rome, Viale Regina Elena 324, 00161 Rome, Italy

**Keywords:** Well-differentiated thyroid carcinoma (WDTC), Locally advanced thyroid cancer, Extra thyroid extension (ETE), AJCC 8th edition Cancer Staging Manual

## Abstract

**Background:**

Well-differentiated thyroid carcinoma is defined as locally advanced in the presence of an extra thyroid extension, e.g., when the surrounding structures such as the trachea, larynx, esophagus and main blood vessels are invaded by cancer. The 8th edition AJCC Cancer Staging Manual states that this is the main characteristic to evaluate for the staging and consequently for the prognosis in patients over 55 years old.

**Main body:**

Distinguishing different forms of locally advanced thyroid cancer is essential, and the various anatomical structures and the clinical and therapeutic consequences must be taken into account. An accurate diagnosis of the organs invaded by thyroid cancer is necessary for the planning of surgical treatment, and both aspects are crucial to improving the patients’ survival. Patients affected by thyroid cancer with extra thyroid extension have a poor prognosis and the removal of the entire neoplasm represents a key factor for better disease-free survival.

**Conclusions:**

We discuss the changes introduced by the 8th edition AJCC Cancer Staging Manual, in terms of the diagnostic and surgical management of extra thyroid extension, in patients affected by papillary and follicular thyroid cancer.

## Background

The AJCC has in recent years completely revised the thyroid cancer staging in its 8th edition, leading to a significant change in the stadiation and treatment of patients with thyroid cancer. The main innovations in stadiation are in terms of attributing a different value to the patient’s age and tumor extension and lymph nodal involvement. The 7th edition only included patients younger than 45 in its better prognostic classification, which exclusively considered the presence/absence of distant metastasis (stages I/II) with better survival rates (98–100%, 85–95%, respectively), while patients older than 45 were classified as having a poor prognosis (stages I to IV, based on TNM). In the 8th edition, the age of inclusion in the classification of a poor prognosis has been changed from 45 to 55 years. A few preliminary remarks are required here about stadiation based on tumor extension and lymph nodal involvement (of patients over 55). Well-differentiated thyroid carcinoma (WDTC) comprises the majority (> 90%) of thyroid cancers, with 10–15% presenting as locally advanced [1]. The WDTC is defined as “locally advanced” in the presence of an extra thyroid extension (ETE), i.e., when the primary tumor passes the thyroid capsule infiltrating the surrounding tissues. The ETE involves several structures such as strap muscles and the trachea, larynx, esophagus, and superior and inferior laryngeal nerves eventually infiltrating the vascular structures or the prevertebral fascia. The different forms of locally advanced thyroid cancer must be distinguished, depending on the anatomical structures involved, which will influence both clinical presentation and therapeutic consequences. At present, the American Joint Committee on Cancer (AJCC) classifies a tumor of any size that has grown extensively beyond the thyroid gland into the nearby anterolateral structures of the neck, such as the larynx, trachea, esophagus or laryngeal nerves, as T4a. The AJCC considers any size of cancer that has grown backward beyond the thyroid gland and involving the spine or the large neck blood vessels as “very locally advanced” (T4b). Particular significance has recently been attributed to ETE in the new 8th edition AJCC Cancer Staging Manual, as having a major effect on different stage-related specific survival rates [2].

## Comparison between 7th and 8th AJCC editions thyroid Cancer Staging Manual

By comparing the two classifications it emerges that in the new edition, the leading factor for early stages (I, II) is represented by the involvement of the lymph node, while the main influencing factor in advanced stages (III, IV) is the ETE (Table 1). In the 7th edition the transition from stage I to stage II is determined exclusively by tumor size, while in the 8th edition this transition is determined by lymph nodal involvement regardless of size. In addition, in the 7th edition stage III exclusively included patients with ETE invading the strap muscles (sternohyoid, sternothyroid or omohyoid muscles) or with lymph nodal metastases to level VI (N1a, i.e., pretracheal, paratracheal and prelaryngeal/Delphinian lymph nodes). In the new evaluation in the 8th edition, stage III includes patients with gross ETE in major structures of the neck (T4a), regardless of lymph nodal involvement. Furthermore, stage IVa in the 7th edition included all patients with gross ETE (T4a) or with lymph node metastasis (N1a or N1b, i.e., the laterocervical lymph node), while in the 8th edition the same stage (IVa, i.e., the involvement of anterolateral structures of the neck), is determined by tumor ETE classified as T4b (involvement of spine and/or large vessels). In the 7th edition stage IVb included tumors classified as T4b regardless of lymph nodal or distant metastasis, while in the current edition the only factor of transition from stage IVa to IVb is represented by distant metastasis. In the latest 8th classification, stage IVc has been removed. The differences in lymph nodal involvement and sites between the two classifications is relevant. In the 8th edition, the presence of lymph nodes metastasis is considered in a single category (N1), regardless of the anatomical site of the lymph node (central or laterocervical). Thus, according to the 8th edition in early-stage tumors lymph nodal involvement appears crucial for stage transition regardless of the site, while in advanced-stage tumors (stage III/V) the evaluation of the ETE is critical and influences survival rate independently from lymph node metastasis. In summary, the analysis of this new classification reveals that the prognostic factors considered unfavorable for survival are over 55 years of age, lymph nodal involvement, ETE, and distant metastases at diagnosis.

**Table 1:**
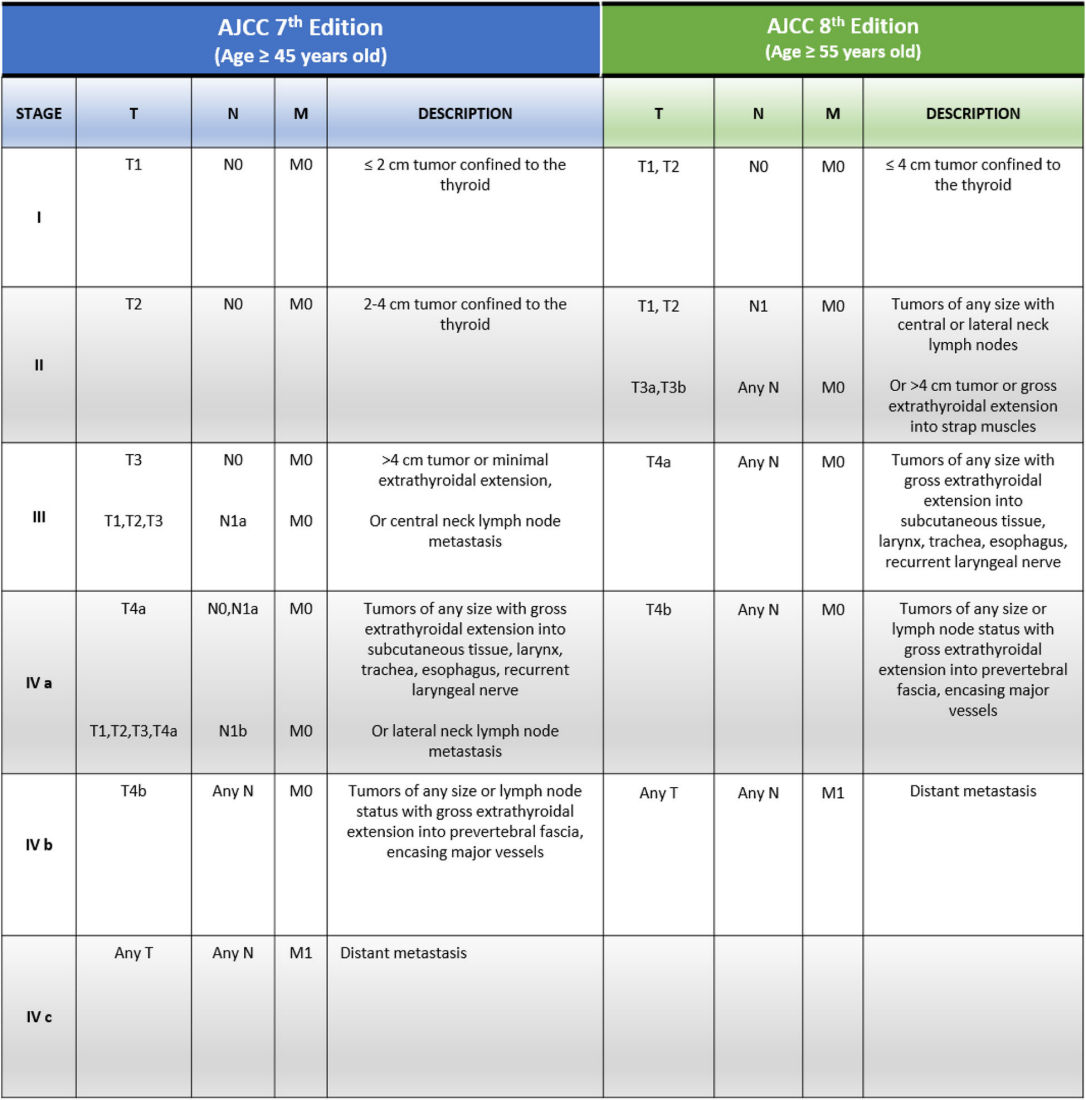
Comparison between 7th and 8th AJCC editions thyroid Cancer Staging Manual

In the 8th edition the definition of T3 has been revised (T3a tumors are more than 4 cm in the maximum dimension, limited to thyroid and T3b tumors of any size with gross extrathyroidal extension into the strap muscles). The difference between central and laterocervical neck lymph nodes and if stage IVc is not present is not considered. The age for poor prognosis has also changed from 45 to 55 years old

## Prevalence of ETE in WDCT

The prevalence of ETE in WDCT has been known for many years. McCaffrey et al. described the ETE of WDCT in 262 patients treated over a 60-year period at the Mayo Clinic, and found that the sites of invasion were the muscle (53%), recurrent laryngeal nerve (47%), trachea (37%), esophagus (21%), and larynx (12%) [3]. These results are comparable with those of a recent study showing that the most common sites of ETE were the recurrent laryngeal nerve (51.0%) followed by the trachea (46.4%) and esophagus (39.2%) [4]. In terms of surgical technique, the neoplastic infiltration of the strap muscles alone, even if it indicates a minimal locally advanced lesion, is not a particular problem. However, the therapeutic approach in a case of invasion of the esophagus and trachea or inferior laryngeal nerve is still a matter of debate [4, 5]. Patients with ETE are known to have a poor prognosis, and according to various authors, the best treatment consists of the removal of the entire neoplasm for better disease-free survival [6]. The aim of radical surgery is not only to eliminate cancer but, when possible, to preserve the functionality of the anatomical structures involved. The conservative surgery aimed at removing only the tumor from the trachea, which is associated with radioiodiotherapy or external radiotherapy is rarely effective if a macroscopic disease residue persists, and there is evidence that this incomplete exeresis further worsens the prognosis [7, 8].

## ETE in the airways

The larynx is affected by direct infiltration of the tumor or by metastatic lymph nodes in the extracapsular space. Laryngeal infiltration affects, in order of frequency, cricoid and thyroid cartilages, and it is often associated with recurrent nerve paralysis due to the simultaneous involvement of a lower laryngeal nerve [9]. The infiltration of the trachea by direct diffusion occurs through the intercartilaginous spaces, a locus minoris resistentiae, in which the submucosa tracheal communicates with the pretracheal fascia [10]. The entire circumference of the trachea may be involved, but the anterior or anterolateral tracts are usually the most affected. In the classification proposed by Shin et al. [11] the tracheal invasion from papillary carcinoma is separated into 4 stages according to the depth of the invasion. Stage 1: carcinoma is limited to the glandular parenchyma; Stage 2: invasion of the cartilage of tracheal rings or intercartilaginous tissue; Stage 3: invasion of the lamina propria of the tracheal mucosa with no elevation or penetration of the mucosa; and Stage 4: full-thickness invasion of the trachea with ulcerations or neoplastic vegetations visible through the bronchoscope. The symptomatology is heterogeneous and concerns the entire involvement of the tracheal, larynx and laryngeal nerves, and up to stage 3 Shin patients are usually asymptomatic. In stage 4 with stenosis, less than 50% present as clinically silent, while over 50% present dyspnea, cornage, triage (for the compensation by the accessory inspiratory muscles and in particular the sternocleidomastoid muscles), and respiratory crisis due to the difficult expectoration of stagnant secretions downstream of the stenosis. Other possible signs and symptoms are hemoptysis, cough, dysphonia, cyanosis and acid-base disorders (the last two present only in the asphyxia phase). Ultrasonography is the most sensitive diagnostic method for the preoperative diagnosis of adventitia or intercartilaginous invasion, and is superior to CT scans and MRI [12]. Laryngeal infiltration is generally asymptomatic if not associated with recurrent paralysis, clinical signs related to the obstruction of the respiratory space, laryngeal competence, and above or subglottic infiltration can be considered exceptional [13, 14].

## ETE in the digestive tract

Secondary neoplastic involvement of the esophagus is infrequent and is usually due to lymph node metastasis in the extracapsular space, or direct involvement (as recurrence after exeresis or in cases of posterior neoplasms). The invasion of the digestive tract frequently occurs simultaneously with the involvement of the lower laryngeal nerve and the respiratory tract [15]. An evaluation of the depth of parietal infiltration is fundamental to establishing a conservative or demolitive surgical approach. Demolitive surgery can significantly worsen the patients’ quality of life, so the surgical reconstruction of the trachea and esophagus poses a major challenge to surgeons. Echoendoscopy can be used to evaluate the extent of esophagus-laryngeal invasion and improve the evaluation of the involvement of the muscular tunic, with a specificity and diagnostic accuracy of 82.9 and 82.7%, respectively. In comparison, MRI results in 60 and 82.7%, respectively, and X-ray esophagograms 58.8 and 60% [16–19]. The most effective method for evaluating intramucosal infiltration has been suggested to be bronchoscopy associated with superficial ultrasound, to examine the infiltration of the adventitia and the intercartilaginous invasion, which is necessary to evaluate the removal of the tracheal rings [20].

## Concerning vascular structures

The internal jugular vein is the most frequently involved vascular structure, and is affected in about 21% of cases. Other vascular structures often involved are the brachiocephalic vein in 9% and the common carotid in 6% [21]. The literature reports cases of neoplastic thrombus of the internal and external jugular veins [22] and internal bilateral internal resection with the reconstruction of the internal jugular vein [23].

## Surgical management of locally advanced WDTC

A desirable curative result is expected from the complete exeresis of the neoplasia together with the infiltrated structures and an adequate lymphectomy. The association of radiotherapy can be curative in selected cases and is relatively efficient in terms of survival and long-term palliation. In case of incomplete exeresis, in the presence of a locally advanced cancer with ETE that compromises the functionality of the surrounding organs, it is advisable to proceed with palliative treatment, aimed at maintaining the patency of the respiratory lumen.

## Airways involvement

In the case of trachea involvement, the exeresis includes total thyroidectomy in a block, with tracheal resection (up to 4–5 tracheal rings) and anastomosis with the inferior part of the cricoid [24]. In the case of laryngeal extension pharyngolaryngectomy, total laryngectomy or hemy-laryngectomy can be performed followed by reconstruction with regional flaps (pectoral/Bakamjian flaps, gastric pull-through procedures or the jejunum free flap) [25]. In these cases, laryngeal paralysis is often already present. In cases of deeper involvement of the hemylarynge, the resection may extend to include part of the vocal cord. It is advisable to perform a complementary tracheotomy downstream of the anastomosis, which will be closed in the immediate postoperative period after the assessment of an adequate residual glottic space. In rare circumstances a microscopically positive laryngeal resection margin is acceptable to preserve laryngeal function, and programming radial treatment after the consolidation of the anastomosis.

## Resection of the esophagus

Resection of the esophagus is rare and is generally performed only in association with airway resection. If the ETE involves only the external muscular layers, it is possible to surgically remove the infiltrated portion, thus saving the submucosa. In a case of full-thickness esophagus involvement the resection of the tract involved is necessary, followed by reconstruction through a cervical esophagus-gastro-plastic or, rarely, esophagus-colon cervical plastic. In some cases these procedures can be performed during the “cervical-mediastinal exenteration”, which involves the laryngeal exeresis of the upper trachea, the resection of the sternal handlebars and the execution of a mediastinal tracheostomy. Surgical exeresis is in these cases is an optional procedure dictated by the local situation, as it considerably aggravates the already notable surgical trauma and postoperative morbidity.

## Surgical reconstruction of the esophagus and trachea

Lesions involving both the esophagus and the trachea are frequently considered nonresectable because of the difficulties in reconstructing these combined defects. In addition, patients eligible for this kind of surgery have usually undergone chemotherapy or radiotherapy, which can compromise the wound healing process. Thus, tracheal and esophageal lesions represent a major challenge to surgeons. However, in the case of a large tracheal or esophageal infiltration, reconstruction is possible with free flaps using microvascular anastomosis. Many surgical techniques for these types of reconstructions have been described in the literature, such as using the myofascial/myocutaneous pedicled flap, or free tissue transfer of a fasciocutaneous flap or jejunum. The various flap-based reconstruction procedures include free bipaddled anterolateral thigh flaps, free radial forearm flaps, and the free posterior tibial artery perforator flap [26–28]. The surgical research in this field is dynamic as the implications a valid and long-term reconstruction of trachea and esophagus have on the patient’s quality of life must be considered. A novel surgical technique was recently proposed for simultaneous tracheal and esophageal reconstruction, using a free bipaddled posterior tibial artery perforator [29]. The authors suggest that this technique has various advantages in cases of the simultaneous reconstruction of two large defects (the trachea and esophagous), such as one perforator artery supplying two flaps as for the bipaddled anterolateral thigh flap, and results in better tissue properties such as those in the free posterior tibial artery perforator flap. The technique may also have advantages in the simultaneous reconstruction of two organs such as the trachea and esophagus. Vascularization is improved as one perforator artery supplies two flaps, as for the bipaddled anterolateral thigh flap, and results in better quality tissue, such as those in the free posterior tibial artery perforator flap.

## Endoscopic treatment

Endoscopic treatment has palliative significance and is reserved for advanced cases of infiltration of the air-digestive pathway from unresectable tumors. The indication of this intervention is generally respiratory problems and dysphagia. Interventional bronchoscopy, including Nd-YAG laser and airways stenting, is an alternative to surgery in inoperable thyroid-induced tracheal obstruction [30, 31].

## Conclusions

The extension of the tumor into the adjacent tissues or organs in WDTC is considered the most important adverse prognostic factor for disease recurrence and in particular for the appearance of distant metastases. However, minimal ETE was defined as an intermediate risk feature in the 2015 American Thyroid Association guidelines [32]. Several studies have suggested that minimal ETE has little effect on disease outcome [33, 34], even if there is no consensus among researchers about the definition of minimal ETE [35]. However, numerous studies have been conducted to identify other prognostic factors that affect mortality and determine the recurrence of the disease in such patients, but have produced contradictory results [36, 37]. As previously described, the prognostic factors assessed with multivariate analysis have shown that an age of above 55 years, the presence of metastases at a distance, and extensive gross extrathyroidal extension are unfavorable factors in the prognosis. Age is also considered a determinant of the response to therapy and disease-specific survival in high-risk thyroid cancer patients. According to Shah and Boucai [38], young patients (age < 55) are much better responders than old patients (age ≥ 55), at 40.3 and 27.5%, respectively, and the proportion of incomplete structural responders was higher in the old group than the young group, with 53 and 33%, respectively. These data show that high-risk young patients with an incomplete structural response to therapy have a significantly better disease-specific survival than older patients (74 and 12%, respectively). For elderly patients, after age stratification it was shown that survival did not appear to be influenced by tumor diameter or incomplete resection, suggesting that a poor prognosis is mainly determined by the patients’ age. These aspects are taken into account in the AJCC’s 8th edition of Cancer Staging, which considers all patients affected by WDTC under 55 years old, any T, any N, and without distant metastases as stage 1, while the presence of metastases is classified as stage 2. There is much evidence that age is the main factor to consider in the staging, independently from the thyroid cancer extension. These concepts represent an innovative change from the previous staging (AJCC’s 7th edition), and the age for poor prognosis has changed from 45 years to 55 years. Shen et al. [39] suggest that the age-associated mortality risk in WDTC is also dependent on BRAF status. According to their findings, age is a strong, continuous and independent mortality risk factor in patients with BRAF V600E mutation. However, for patients over 55 years old, the ETE extension is the central aspect to evaluate in the assignment of the stage. Historically, the prognosis in patients with ETE appears to be influenced by aggressive surgical treatment, which is essential for proper therapy and longer survival. In the case of WDTC invading trachea, Ishihara et al. [40] reported that 3-, 5-, and 10-year survival rates in patients who underwent complete resection were 87.0, 78.1, and 78.1%, respectively. Conversely, for patients who underwent incomplete resection, the survival rates were 64.9, 43.7, and 24.3%, respectively. A retrospective study performed by McCarty et al. [41] to determine the results of laryngotracheal resection or tracheal cartilage shave with adjuvant radiotherapy showed that the rates of 10-year disease-free survival and overall survival for all patients were 47.9 and 83.9%, respectively. The indication for tracheotomy, salvage procedures, or supportive care has decreased over time, notwithstanding that some patients continue to present ETE. In conclusion, it must be highlighted that locally advanced thyroid tumors have an incidence correlated with increasing age, and are frequently associated with more aggressive histological forms, such as papillary variants in sclerosis and high cells.

## Data Availability

The data supporting the conclusions of this paper are included in the article.

## Abbreviations

AJCC: American Joint Committee on Cancer
ETE: Extra thyroid extension
WDTC: Well-differentiated thyroid carcinoma
